# The use of denial in an ethnically diverse British cancer population: a cross-sectional study

**DOI:** 10.1038/sj.bjc.6602523

**Published:** 2005-04-05

**Authors:** R Roy, R P Symonds, D M Kumar, K Ibrahim, A Mitchell, L Fallowfield

**Affiliations:** 1 Department of Oncology, Leicester Royal Infirmary, Leicester LE1 5WW, UK; 2 Department of Cancer Studies & Molecular Medicine, University of Leicester, Osborne Building, Leicester Royal Infirmary, Leicester LE1 5WW, UK; 3 Brandon Mental Health Unit, Leicester General Hospital, Leicester LE5 4PW, UK; 4 CRUK Psychosocial Oncology Group, University of Sussex, Sussex BN1 9QG, UK

**Keywords:** denial, anxiety, depression, ethnicity

## Abstract

A total of 82 Asian and 117 randomly selected white Caucasian patients at the Leicestershire Cancer Centre were assessed using measures of coping and adaption to cancer. On the Mental Adjustment to Cancer (MAC) scale, Asian patients were more fatalistic (*P*<0.0001) and had more significant hopeless/helpless scores (*P*=0.007). The two ethnic groups answered the three questions thought to assess denial differently. Caucasians were more likely not to dwell on their illness (73 *vs* 55.5%, *P*<0.0001) and agree with the statement ‘I have difficulty believing this is happening to me’ (73 *vs* 60.5%, *P*<0.0001). However, Asian patients were more likely to agree with the statement ‘I don't really believe I have cancer’ (48.2 *vs* 31.3%, *P*=0.019). Within both groups there was an association with denial and anxious preoccupation (*P*<0.001). On the Hospital Anxiety and Depression (HAD) scale, there was no difference in anxiety scores between either sexes or between the Asian and Caucasian groups. However, Asian patients were more depressed (*P*=0.001). Although denial was significantly related to the presence of both depression (*P*<0.0001) and anxiety (*P*=0.001) in the entire patient population, there were different predictors of denial in each subgroup. On multiple regression analysis depression was linked with denial in Caucasians, whereas Fighting Spirit (minus helplessness/hopelessness) was linked with denial in Asian patients. There are definite differences in coping styles in British cancer patients according to ethnicity. While significant numbers in both groups employ denial in some form, Caucasian patients appear to adapt to the psychological pressures of cancer more successfully than Asian patients at a particular point in time. Further work is required to elucidate longitudinal relationships between denial and adaption to cancer.

Over the last two decades survival rates following cancer have improved in the UK not only for uncommon cancers such as testicular tumours and lymphomas but also for common cancers such as breast or prostate (Boyle *et al*, 2003; Levi *et al*, 2004). In parallel with the improvements in survival, there has been a greater emphasis on improving the quality of life of patients (Brown *et al*, 2000). Psychological and psychiatric symptoms have a detrimental impact upon quality of life (Shapiro *et al*, 2001) and yet tend to be overlooked in busy clinical environments (Fallowfield *et al*, 2001; Parle *et al*, 2001). One reason for this under-recognition may be that some patients do not feel able to volunteer symptoms of distress to medical staff within a relatively short clinical interview. This is most likely in patients who attempt to minimise the significance of the impact of their diagnosis. Such patients are often described as using the coping strategy of denial.

We have previously shown that different ethnic groups have different information needs in relation to cancer (Kumar *et al*, 2004). Different ethnic groups may well have different coping strategies to deal with psychological pressures following the diagnosis of cancer. Such an effect would be expected to act in concert with other influences on coping style such as social support as well as individual differences. Such differences in coping styles are highly relevant to multi-ethnic communities such as Leicester, Bradford and Birmingham, where British Asians comprise more than a quarter of the population. Prospective studies have shown that differences in coping style influence long-term adaptation to cancer, and in turn influence quality of life (Sehlen *et al*, 2003). Further, in order to offer optimal treatment for psychological as well as physical complications, it is vitally important to understand how ethnically diverse populations react to a diagnosis of cancer.

## MATERIALS AND METHODS

A total of 138 Asian patients who were defined by registered ethnicity details, appearance or name were seen during a 14-month period beginning in August 2001 at the Leicestershire Cancer Centre. Attempts were made to interview as many of these patients as possible. In all, 82 Asian patients and 117 randomly selected Caucasian patients were interviewed as part of a study of patients' information needs, which the local research ethics committee had approved. Fifteen Asian patients declined to be interviewed and the families of five Asian patients refused to allow participation. Sixteen Caucasian patients also declined the invitation. Questionnaires were posted to 10 patients. None of these was returned.

An Oncologist or Radiographer, either in English or in an Asian language including Gujarati, Punjabi, Urdu, Bengali or Hindi, carried out the interviews. Caucasian patients had an equal opportunity to complete the questionnaires on their own or guided by an interviewer. All patients who took part in the study were given their questionnaires in a treatment clinic (radiotherapy/chemotherapy) and these were completed and returned on the same day. Prior to entering the study, all patients were told of their diagnosis firstly by the oncologist and then by either the chemotherapy nurse or the therapy radiographer. All patients signed an initial consent form for cancer treatment and a further consent form for this study, which contained the word ‘cancer’. There was a second reminder of their original diagnosis by virtue of signing the consent form for the study. Seventy-three Asian patients were conversant enough in English to carry out the consultation in the same language. Of the nine remaining, seven had their diagnosis interpreted in Gujarati and two in Urdu by a member of the staff. It is therefore reasonable to conclude that all patients were factually aware of their diagnosis.

Patients completed the Mental Adjustment to Cancer (MAC) scale to assess the coping style and the Hospital Anxiety and Depression (HAD) to assess adaptation to cancer. Both are well-validated tools used to measure coping and distress in cancer patients. The MAC is a 40-item questionnaire, which incorporates five subscales to measure fighting spirit, helpless/hopelessness, anxious preoccupation, fatalism and avoidance/denial (Watson *et al*, 1988). Recent work suggests that the construct of denial is addressed by three questions (questions 5, 21 and 38) on the MAC scale rather than the originally proposed single item (question 38) (Nordin *et al*, 1999). The responses ‘Applies to me’ (score=3) and ‘Definitely applies to me’ (score=4) were combined in the analysis.

Although the original version of the MAC scale had only been validated in UK population (Watson *et al*, 1988), it has undergone assessment and validation among patient populations of other ethnicities and countries (Akechi *et al*, 2000; Cayrou *et al*, 2003; Ho *et al*, 2003; Mystakidou *et al*, 2004). In each case the internal consistency was found to be similar to that of the original version and concurrent validity and correlation was established with other frequently used tools. The HAD scale has also been validated in cancer patients (Carroll *et al*, 1993). As the MAC scale had not been validated in either Gujarati or Urdu and the HAD scale had only been validated in Urdu, we analysed the results separately for patients who had the questionnaires translated to them in their mother language.

SPSS 11 software was used for the statistical analysis. All variables were tested for normality using the Kolomogorov–Smirnoff test and attempts were made to convert them to normal distribution by established methods. Variables that could be recoded into categorical variables (e.g., anxiety, depression, ethnicity) were tested using *χ*^2^ tests and the rest were analysed using the Mann–Whitney test statistic. Multiple regression analyses were carried out, with each variable entered in a stepwise manner.

## RESULTS

The major clinical and demographic features of the two groups are listed in Table 1. There was a trend for Asian patients to be younger, reflecting the younger mean age of the Asian community, but the difference was not significant (*P*=0.164). There was a statistically significant greater proportion of female patients in the Caucasian group (*P*=0.047). The number of patients receiving radical (potentially curative) or palliative treatments was similar in both groups. Data were not collected separately for educational levels and socio-economic status of individual patients. The mean time from diagnosis to interview for Caucasian patients was 20.1 weeks (range 0–45, s.d. 9.37) and for Asian patients 15.4 weeks (range 0–45, s.d. 9.03). For patients presenting with metastatic disease, the interval was calculated from the time of presenting with either clinically or radiologically proven metastasis.

### Use of coping styles (the MAC Scale) (Table 2)

There was no difference in fighting spirit between the two cultural groups (*P*=0.179). On the other hand, Asian patients were more fatalistic (*P*<0.0001) and had more significant hopeless/helpless scores (*P*=0.007). Excluding the nine non-English speaking patients for whom the scale had been interpreted to Gujarati and Urdu, the results for these factors did not change. However, Asian patients who were English speaking showed significantly higher levels of anxious preoccupation compared with Caucasian patients (*P*=0.014), an effect not seen when English- and non-English-speaking subgroups were combined.

Regarding denial, the two ethnic groups answered the three denial questions differently (Table 3). In response to Question 5 – ‘I do not dwell on my illness’, Caucasian patients were more likely to agree than their Asian counterparts (73 *vs* 55.5%, *P*<0.0001) and also concur with the statement ‘I have difficulty believing that this happened to me’ (Question 21) (73 *vs* 60.5%, *P*<0.0001). However, Asian patients were far more likely to agree with the statement ‘I don't really believe I have cancer’ (Question 38) (48.2 *vs* 31.3%, *P*=0.019). If the scores of the nine patients were excluded from the analysis, the results still remained significant. Across all patients there was an association between denial (on question 38) and the presence of anxious preoccupation (Spearman's correlation coefficient =0.233, *P*=0.001), which remained significant in both Caucasians alone (*P*<0.028) and British Asians alone (*P*<0.0271) in univariate analysis.

### Predictors of the use of denial (the MAC Scale) (Tables 4 and 5)

Using question 38 as the outcome variable, we calculated the ‘best fit’ predictors using multiple regression analysis and entering age at study entry, time from diagnosis, level of anxiety, level of depression and the remaining three coping styles for both Asian and Caucasian patients. Among Caucasian patients, the only significant predictor of use of denial was depression when all other predictors were entered into the equation. Among Asian patients, the only significant predictor was fighting spirit (strictly adjusted fighting spirit minus helplessness/hopelessness scores), with age (*P*<0.054), anxious preoccupation (*P*<0.077) and fatalism (*P*<0.058) showing a trend and no effect from depression and anxiety.

### Adaptation to cancer (the HAD Scale) (Table 2)

There was no significant difference in anxiety scores between either sexes or between the Asian and Caucasian groups. However, Asian patients were more depressed (20.7 *vs* 10.4%, *P*=0.001) using a cutoff score of 10 in the HAD scale. Using a higher cutoff of 13 (Smith *et al*, 2002), 7.3% Asians were found to fulfil the criteria for significant depression compared to 1% of Caucasians (*P*=0.021). There was a nonsignificant trend for Asian women to be more depressed than female Caucasians (51.1 *vs* 34.9%, *P*=0.072). Both depression and anxiety were significantly related to the presence of denial (Question 38) (correlation coefficient=0.317, *P*<0.0001 and correlation coefficient=0.232, *P*=0.001, respectively) when calculated in the entire patient population, but not in Asian patients considered alone.

## DISCUSSION

While previous studies have reported ethnic differences in coping styles including denial (Culver *et al*, 2002), this is the first study to examine the British Asian community. Asian patients tend to be more fatalistic than white Caucasians and are more likely to feel more helpless. The use of denial was also distinct in the two ethnic groups. A higher proportion of Caucasians cope by not dwelling on their illness. Although Asian patients were clearly told that they had cancer and signed at least one consent form for cancer treatment, 48.2% said that they did not really believe they had cancer. This illustrates that the concept of denial is multifaceted. Previous studies have shown that those individuals who do not dwell on their illness and have less intrusive thoughts of cancer tend to achieve better long-term adaptation and better quality of life (Hack and Degner, 2004; Kershaw *et al*, 2004). Our cross-sectional study supports this association, but also suggests that this process is more likely in Caucasian patients who used denial less often and were less depressed. On the other hand, those patients who have difficulty in believing their diagnosis may have more intrusive thoughts about their disease and feel more helpless or anxious (Primo *et al*, 2000). These individuals tend to do less well in the long term, with higher rates of depression and anxiety and poorer quality of life. In our study, Asian patients used denial more often and were more likely to be depressed. In univariate analysis, anxious preoccupation (and to a lesser extent syndromal anxiety) was associated with the use of denial. However, when other factors were controlled for in multivariate analysis, this effect was reduced to a trend in both subgroups. Depression was the most significant variable linked with the use of denial, but only in Caucasian patients. In Asian patients fighting spirit minus helplessness/hopelessness was inversely linked with denial. It is quite possible that these measures are facets of the same dimension that is low mood because of the way FSH is calculated from raw scores. This can only be resolved by longitudinal follow-up. It is likely that use of adaptation and coping will change with time during the individual's journey through cancer.

The differing coping styles and adaptation of the two ethnic groups could be influenced by their different socio-economic status and educational levels. We did not set out on this study to segregate patients on that basis, but elected to have a random sample with the aim of reducing any bias. Although in many regions within UK the majority of Asian families fall into low-income groups, this is not the case in Leicestershire. Data from the 1991 census show very similar levels of affluence in the Asian and Caucasian communities in the county (http://www.empho.org.uk/products/ethnicity/appendix2.doc). However, we cannot rule out the possibility that differences in socio-economic status and education may have affected the results in this study. Other limitations of this study are the modest sample size and the fewer number of patients receiving palliative treatment in both ethnic groups. This is due to the fact that patients treated with palliative intent often completed their treatment before the ethnicity details were registered. A further area of scrutiny is the documentation of exactly what patients understood about their diagnosis. Although we were able to standardise what patients were told about their illness, we are aware from previous studies that there are individual differences in how much information patients retain (Thomas *et al*, 1999). Whether there are significant differences between ethnic groups remains to be shown. Regarding the issue of language, analysis without nine patients (seven Gujarati and two Urdu) that relied upon an interpreter had a subtle but statistically significant effect on the proportion rated as having ‘anxious preoccupation’, but no other variables. This may suggest that interpreters underestimate the degree of anxious pre-occupation in subjects, an area for further study.

In summary, there are definite differences in coping style in British cancer patients according to ethnicity when interviewed at one point in time. While significant numbers in both groups employ denial in some form, for reasons that are as yet unclear in Caucasian patients, relatively few had developed depression compared to Asian patients at the time of our assessment. Intriguingly, predictors of denial differed between groups, which may suggest divergent long-term trajectories in adaption. In particular, it will be of interest to discover what are the risk factors for long-term morbidity in Asian and Caucasian patients, and this will be a part of further studies planned by our group.

## Figures and Tables

**Table 1:** Demographic and clinical details (percentages in brackets)

	**British Asians**	**White Caucasians**
Number	82	117
Age	51.96 (18–77)	61.92 (33–89)
	*P*=0.164	
Male	35 (42.6)	34 (29.1)
Female	47 (57.3)	83 (70.9)
	*P*=0.047	
		
*Tumour site*		
Breast	32 (39.0)	50 (42.7)
Prostate	11 (13.4)	12 (10.2)
Colon	14 (17.0)	10 (8.5)
Cervix/uterus	8 (10.0)	7 (6.0)
Other	17 (20.6)	38 (32.6)
		
*Type of treatment*		
Radical	71 (86.5)	98 (83.7)
Palliative	11 (15.0)	19 (16.2)

**Table 2:** The MAC and HAD Scale summary

**Traits**		**Patients employing a trait (%)**	**Mean score (s.d.)**	**Mean score (s.d.) for Asian patients using English only**	**Overall *P*-value (*P*-value for Asian patients using English only)**
Fatalism (eight items of the MAC Scale)	Caucasian	50.4	48.66 (7.94)	48.66 (7.94)	*P*<0.0001 (*P*<0.0001)
	Asian	84.9	57.64 (11.2)	57.53 (11.72)	
Anxious Preoccupation (nine items of the MAC Scale)	Caucasian	75.7	53.79 (8.1)	53.79 (8.1)	*P*=0.142 (*P*=0.014)
	Asian	78.1	55.57 (14.51)	57.12 (14.08)	
Hopelessness (six items of the MAC Scale)	Caucasian	42.6	10.52 (3.03)	10.52 (3.03)	*P*=0.007 (*P*=0.004)
	Asian	53.4	11.84 (3.47)	11.96 (3.55)	
Fighting Spirit (16 items of the MAC Scale)	Caucasian	71.3	47.67 (8.33)	47.67 (8.33)	*P*=0.179 (*P*=0.974)
	Asian	80.8	48.64 (7.21)	48.44 (7.52)	
Anxiety	Caucasian	12.2	6.17 (3.85)	6.17 (3.85)	*P*=0.257 (*P*=0.301)
	Asian	20.7	6.94 (4.41)	7.14 (4.17)	
Depression					
	Caucasian	10.4	5.11 (4.00)	5.11 (4.00)	*P*=0.001 (*P*=0.001)
	Asian	20.7	7.43 (5.04)	7.71 (4.89)	

**Table 3:** Denial question (percentages in brackets)

**Question**		**1 Definitely does not apply to me**	**2 Does not apply to me**	**3 Applies to me**	**4 Definitely applies to me**	**P value for all patients (3+4 combined)**	**P value for Asian patients using English only**
I don't dwell on my illness (Question 5)	Caucasian	4 (3.5)	27 (23.5)	56 (48.7)	28 (24.3)	*P*<0.0001	*P*=0.001
	Asian	19 (23.5)	17 (21.0)	36 (44.4)	9 (11.1)		
I have difficulty believing that this happened to me (Question 21)	Caucasian	9 (7.8)	22 (19.1)	69 (60.0)	15 (13.0)	*P*<0.0001	*P*<0.0001
	Asian	15 (18.5)	17 (21.0)	23 (28.4)	26 (32.1)		
I don't really believe I have cancer (Question 38)	Caucasian	27 (23.5)	52 (45.2)	31 (27.0)	5 (4.3)	*P*=0.019	*P*=0.046
	Asian	16 (19.8)	26 (32.1)	25 (30.9)	14 (17.3)		

**Table 4:** Multiple regression of predictors of denial in Caucasian patients

Multiple *R*=0.426					
*R^2^*=0.181					
Adjusted *R^2^*=0.127					
					
**Predictors**	**Coefficients**	**Lower 95%**	**Upper 95%**	***t* Stat**	***P*-value**
Age	−0.001	−0.016	0.014	−0.158	0.875
Time since diagnosis	0.012	−0.005	0.028	1.417	0.159
Anxious preoccupation	0.012	−0.010	0.034	1.081	0.282
Fatalism	0.007	−0.012	0.026	0.713	0.477
Fighting spirit – Helplessness/Hopelessness	0.007	−0.008	0.023	0.955	0.342
Anxiety	−0.007	−0.058	0.044	−0.276	0.783
Depression	0.057	0.003	0.111	2.097	0.038

**Table 5:** Multiple regression of predictors of denial in Asian patients

Multiple *R*=0.512					
*R^2^*=0.262					
Adjusted *R^2^*=0.191					
					
**Predictors**	**Coefficients**	**Lower 95%**	**Upper 95%**	***t* Stat**	***P*-value**
Age	−0.014	−0.030	0.001	−1.953	0.054
Time since diagnosis	0.003	−0.019	0.026	0.30	0.757
Anxious preoccupation	0.016	−0.001	0.034	1.79	0.077
Fatalism	−0.019	−0.040	0.001	−1.92	0.058
Fighting spirit – Helplessness/Hopelessness	−0.025	−0.043	−0.01	−2.82	0.006
Anxiety	0.015	−0.045	0.077	0.50	0.61
Depression	−0.005	−0.05	0.043	−0.224	0.82

## References

[bib1] Akechi T, Fukue-Saeki M, Kugaya A, Okamura H, Nishiwaki Y, Yamawaki S, Uchitomi Y (2000) Psychometric properties of the Japanese version of the Mental Adjustment to Cancer (MAC) scale. Psychooncology 9(5): 395–401 11038477 10.1002/1099-1611(200009/10)9:5<395::aid-pon472>3.0.co;2-o

[bib2] Boyle P, d'Onofrio A, Maisonneuve P, Severi G, Robertson C, Tubiana M, Veronesi U (2003) Measuring progress against cancer in Europe: has the 15% decline targeted for 2000 come about? Ann Oncol 14: 1312–1325 12881398 10.1093/annonc/mdg353

[bib3] Brown JE, King MT, Butow PN, Dunn SM, Coates AS (2000) Patterns over time in quality of life, coping and psychological adjustment in late stage melanoma patients: an application of multilevel models. Qual Life Res 9: 75–85 10981208 10.1023/a:1008995814965

[bib4] Carroll B, Kathol R, Noyes R, Wald T (1993) Screening for depression and anxiety in cancer patients using the hospital anxiety and depression scale. Gen Hosp Psychiatry 15: 69–74 8472942 10.1016/0163-8343(93)90099-a

[bib5] Cayrou S, Dickes P, Gauvain-Piquard A, Roge B (2003) The mental adjustment to cancer (MAC) scale: French replication and assessment of positive and negative adjustment dimensions. Psychooncology 12(1): 8–23 12548645 10.1002/pon.634

[bib6] Culver JL, Arena PL, Antoni MH, Carver CS (2002) Coping and distress among women under treatment for early stage breast cancer: comparing African Americans, Hispanics and non-Hispanic whites. Psychooncology 11: 495–504 12476431 10.1002/pon.615

[bib7] Fallowfield L, Ratcliffe D, Jenkins V, Saul J (2001) Psychiatric morbidity and its recognition in patients with cancer. Br J Cancer 84: 1011–1015 11308246 10.1054/bjoc.2001.1724 PMC2363864

[bib8] Hack TF, Degner LF (2004) Coping responses following breast cancer diagnosis predict psychological adjustment three years later. Psychooncology 13: 235–247 15054728 10.1002/pon.739

[bib9] Ho SM, Fung WK, Chan CL, Watson M, Tsui YK (2003) Psychometric properties of the Chinese version of the Mini-Mental Adjustment to Cancer (MINI-MAC) scale. Psychooncology 12(6): 547–556 12923795 10.1002/pon.672

[bib10] http://www.empho.org.uk/products/ethnicity/appendix2.doc

[bib11] Kershaw T, Northouse L, Kritpracha C, Schafenacker A, Mood D (2004) Coping strategies and quality of life in women with advanced breast cancer and their family caregivers. Psychol Health 19: 139–155

[bib12] Kumar DM, Symonds RP, Sundar S, Ibrahim K, Savelyich BSP, Miller E (2004) Information needs of Asian and White British cancer patients and their families in Leicestershire: a cross-sectional survey. Br J Cancer 90: 1474–1478 15083171 10.1038/sj.bjc.6601774 PMC2409694

[bib13] Levi F, Lucchini F, Negri E, Boyle P, La Vecchia C (2004) Cancer mortality in Europe, 1995–1999, and an overview of trends since 1960. Int J Cancer 110: 155–169 15069676 10.1002/ijc.20097

[bib14] Mystakidou K, Watson M, Tsilika E, Parpa E, Primikiri A, Katsouda E, Vlahos L (2004) Psychometric analyses of the Mental Adjustment to Cancer (MAC) scale in a Greek palliative care unit. Psychooncology Mar 3, (Epub ahead of print) 10.1002/pon.801 15386795

[bib15] Nordin K, Berglund G, Terje I, Glimelius B (1999) The mental adjustment to cancer scale – a psychometric analysis and the concept of coping. Psychooncology 8: 250–259 10390737 10.1002/(SICI)1099-1611(199905/06)8:3<250::AID-PON379>3.0.CO;2-J

[bib16] Parle M, Gallagher J, Gray C, Akers G, Liebert B (2001) From evidence to practice: factors affecting the specialist breast nurse's detection of psychological morbidity in women with breast cancer. Psychooncology 10: 503–510 11747062 10.1002/pon.541

[bib17] Primo K, Compas BE, Oppedisano G, Howell DC, Epping-Jordan JE, Krag DN (2000) Intrusive thoughts and avoidance in breast cancer: individual differences and association with psychological distress. Psychol Health 14: 1141–1153 22175267 10.1080/08870440008407372

[bib18] Sehlen S, Song R, Fahmuller H, Herschbach P, Lenk M, Hollenhorst H, Schymura B, Aydemir U, Duhnke E (2003) Coping of cancer patients during and after radiotherapy – a follow-up of 2 years. Onkologie 26: 557–563 14709930 10.1159/000074151

[bib19] Shapiro SL, Lopez AM, Schwartz GE, Bootzin R, Figueredo AJ, Braden CJ, Kurker SF (2001) Quality of life and breast cancer: relationship to psychosocial variables. J Clin Psychol 56: 501–519 10.1002/jclp.1026 11255204

[bib20] Smith AB, Selby PJ, Velokova G, Stark D, Wright EP, Gould A, Cull A (2002) Factor analysis of the Hospital Anxiety and Depression Scale from a lung cancer population. Psychol Psychotherapy 75: 165–176 10.1348/147608302169625 12396762

[bib21] Thomas R, Deary A, Kaminski E, Stockton D, De Zueew N (1999) Patients' preferences for video cassette recorded information: effect of age, sex and ethnic group. Eur J Cancer Care 8(2): 83–86 10.1046/j.1365-2354.1999.00123.x 10476110

[bib22] Watson M, Greer S, Young J, Inayat Q, Burgess C, Robertson B (1988) Development of a questionnaire measure of adjustment to cancer: the MAC scale. Psychol Med 18: 203–209 3363039 10.1017/s0033291700002026

